# Utilization of Recycled Asphalt Concrete with Warm Mix Asphalt and Cost-Benefit Analysis

**DOI:** 10.1371/journal.pone.0116180

**Published:** 2015-01-09

**Authors:** Julide Oner, Burak Sengoz

**Affiliations:** 1 Department of Civil Engineering, Division of Transportation, Dokuz Eylul University, Graduate School of Natural and Applied Sciences, Izmir, Turkey; 2 Department of Civil Engineering, Division of Transportation, Dokuz Eylul University, Izmir, Turkey; VIT University, India

## Abstract

The asphalt paving industries are faced with two major problems. These two important challenges are generated with an increase in demand for environmentally friendly paving mixtures and the problem of rapidly rising raw materials. Recycling of reclaimed asphalt pavement (RAP) is a critical necessity to save precious aggregates and reduce the use of costly bitumen. Warm Mix Asphalt (WMA) technology provides not only the option of recycling asphalt pavement at a lower temperature than the temperature maintained in hot mixtures but also encourages the utilization of RAP and therefore saves energy and money. This paper describes the feasibility of utilizing three different WMA additives (organic, chemical and water containing) at recommended contents with different percentages of RAP. The mechanical properties and cost-benefit analysis of WMA containing RAP have been performed and compared with WMA without RAP. The results indicated that, 30%, 10% and 20% can be accepted as an optimum RAP addition related to organic, chemical and water containing additives respectively and organic additive with 30% RAP content has an appreciable increase in tensile strength over the control mix. It was also concluded that the RAP with WMA technology is the ability to reduce final cost compared to HMA and WMA mixtures.

## Introduction

Recycling of bituminous materials has generated considerable discussion and development in the last decade. While it is not a new idea, recent studies appear to be in response to the desire of many countries to reduce their dependency on imported crude oil and the derivative product as bitumen. The current development of recycling is ecologically safe and energy saving from the viewpoint of global environment production [1], [2]. The high cost associated with the petroleum and raw material extraction, has justified scientists to search for new materials with the ability of combining durability and performance at low cost [3]. The use of reclaimed asphalt pavement (RAP) provides an economic method for asphalt construction (cold recycled or hot mix asphalt) [4]. RAP contains both aggregates and bitumen, and hence the using of RAP saves natural resources, money as well as it is eco friendly [5]. Over the years recycling has become one of the most desirable pavement rehabilitation alternatives. Based on continuous accumulation of performance data, field and laboratory evaluations of recycled mixes, it is expected that recycling will continue to be the most attractive rehabilitation technique [6]. The choice of rehabilitation technique should be based on energy conservation, economic and engineering consideration, environmental effects.

In recent years, environmental protection is increasingly becoming a major issue in transportation including asphalt production. Despite of the fact that hot mix asphalt (HMA) is widely used around the world, some recent studies suggest using another process that reduces the production and placement temperature of asphalt mixes. There is a new technology is called the warm mix asphalt (WMA), and is used mostly in European countries [7]. The goal of such a mix is to obtain strength and durability that is equivalent to or better than HMA [8]. Currently, a common way of achieving the above mentioned characteristics are through the use of additives. All of the current WMA additives facilitate lowering of production temperature by either lowering the viscosity and/or expanding the volume of the bitumen at a given temperature [9], [10]. By lowering the viscosity or expanding the volume of the bitumen, the aggregates are completely coated in the bitumen at a lower than conventional temperature (approximately 150°C) [11].

WMA technology offers a solution to maintain the current state of technology that enables to utilize RAP at a relatively lower temperature than HMA mixtures. This technology provides a method of attaining low viscosity in the bitumen at relatively low temperatures [4]. O’Sullivan and Wall [11] indicated that the utilization of RAP with WMA technologies decreases the environmental impacts by using less virgin material and reducing CO_2_ emissions. Mallick et al. [12] reported that it is possible to manufacture mixes with RAP with similar properties to HMA mixes through the use of WMA additives.

WMA technology can be classified based on organic as well as chemical additives and the utilization of water. Organic additives are used to improve bitumen flow by reducing viscosity of bitumen [13]. A decrease of viscosity produces asphalt mixtures at low temperatures. After crystallization, organic additives tend to increase the stiffness of the bitumen and asphalt’s resistance against deformations [14]. The different chemical additives are used for particular products. Chemical additives are combination of emulsification agents, polymers and additives to enhance workability, compaction and adhesion. Temperature reduction is provided without addition of water. Chemical additives can encourage the processing of asphalt mixture at lower temperatures with the combination of RAP. The contents of chemical additive used in bitumen were generally based on the recommendations by the suppliers as well as literatures [15]–[18]. Small amounts of water added into the hot bitumen in foaming technology. Injected water evaporates and causes producing large volume of foam. The large volume of foam results in increasing expansion of the bitumen and decreasing the viscosity of bitumen, which improves coating and workability of asphalt pavement mixtures. However; the using of water causes some stripping problems, anti-stripping additives can be used to minimize moisture susceptibility and to provide chemical adhesion between bitumen and aggregate surfaces. When the additive is added to the bitumen and heated together above 57°C to 71°C, 21% of water is released by weight. This foaming action of the liquid bitumen acts as a temporary asphalt volume extender and mixture lubricant, enabling the aggregate particles to be rapidly coated and the mix to be workable and compactable at temperatures significantly lower than HMA [19]. The process used in this research treated the RAP at the contents of 10%, 20%, 30%, 40%, and 50% with WMA additives at recommended contents (organic additive at a dose 3%, chemical additive at a dose 2% and water containing additive at a dose 5% by weight of the bitumen). The mechanical performances of the samples were evaluated by Marshall Stability test and Indirect Tensile Strength test (ITS). Following the experimental studies, cost-benefit analysis was performed to inspect the advantages and disadvantages of RAP in terms of economy.

## Materials and Methods

The base bitumen with a 50/70 penetration grade has been obtained from Aliaga/Izmir Oil Terminal of the Turkish Petroleum Refinery Corporation. In order to characterize the properties of the base bitumen, conventional bitumen tests such as: penetration test (ASTM D5–06), softening point test (ASTM D36–95), thin film oven test (TFOT) (ASTM D1754–97), penetration and softening point after TFOT, etc. were performed [20], [21], [22]. These tests were conducted in conformity with the relevant test methods that are presented in Table 1.

**Table 1:** Properties of the base bitumen.

Test	Specification	Results	Specification limits
Penetration	ASTM D5	55	50–70
(25°C; 0.1 mm)			
Softening Point (°C)	ASTM D36	49.1	46–54
Viscosity at (135°C)-Pa.s	ASTM D4402	0.413	–
Thin Film Oven Test (TFOT) (163°C; 5 hr)	ASTM D1754		
Change of Mass (%)		0.04	0.5 (max)
Retained Penetration after TFOT (%)	ASTM D5	25	–
Softening Point Diff. after TFOT (°C)	ASTM D36	5	7 (max)
Specific Gravity	ASTM D70	1.030	–
Flash Point (°C)	ASTM D92	+260	230 (min)

The asphalt mixtures were produced with limestone aggregates. Fine and coarse limestone aggregates were produced from Dere Beton/Izmir quarry. In order to find out the properties of the limestone aggregate used in this study, sieve analysis (ASTM C136), specific gravity (ASTM C127, ASTM C128), Los Angeles abrasion resistance test (ASTM C131), sodium sulphate soundness test (ASTM C88), fine aggregate angularity test (ASTM C1252) and flat and elongated particles tests (ASTM D4791) were conducted on limestone aggregates [23]–[29]. Grading of aggregate had been chosen in conformity with the Type I Wearing Course of Turkish Specifications. Table 2 presents the properties of the limestone aggregates.

**Table 2:** The properties of limestone aggregates.

Test	Specification	Grading Passing (%)	Specification Limits
***Sieve Analysis***	ASTM C 136		
***Sieve Size/No.***			
3/4″		100	100
1/2″		92	83–100
3/8″		73	70–90
No.4		44.2	40–55
No.10		31	25–38
No.40		12	10–20
No.80		8	6–15
No.200		5.3	4–10
***Specific Gravity (Coarse Agg.)***	ASTM C 127		
Bulk		2.704	–
SSD		2.717	–
Apparent		2.741	–
***Specific Gravity (Fine Agg.)***	ASTM C 128		
Bulk		2.691	–
SSD		2.709	–
Apparent		2.739	–
***Specific Gravity (Filler)***		2.732	–
***Los Angeles Abrasion (%)***	ASTM C 131	22.6	Max. 30
***Flat and Elongated Particles (%)***	ASTM D 4791	7.5	Max. 10
***Sodium Sulfate Soundness (%)***	ASTM C 88	1.47	Max. 10–20
***Fine Aggregate Angularity***	ASTM C 1252	47.85	Min. 40

The organic WMA additive is Sasobit, which is made of Sasol Wax, is a long-chain aliphatic polymethlene hydrocarbon produced from the Fischer-Tropsch (FT) chemical process with a melting temperature of 120°C. The longer chains help to keep the wax in solution which reduces bitumen viscosity at typical asphalt production and compaction temperatures. Based on the available literatures, dosage rates for Sasobit ranged from 1.0% to 4.0% by weight of the bitumen [30], [31], [32]. The organic WMA additive concentration in the base bitumen was chosen as 3.0%. The utilization of this content is based on a past research made by O’Sullivan and Wall [11]. They concluded that Sasobit should be added at a rate of 3.0% by mass of bitumen for maximum effectiveness.

Rediset WMX is a chemical additive that uses a combination of cationic surfactants and organic additive based rheology modifier. Rediset chemically modifies the bitumen and obtains active adhesion force which improves coating of aggregates with bitumen [16]. Rediset can also encourage both processing of asphalt mixture at lower temperatures. Researches indicate that the Rediset should be used at dosage rates at 1.5%, 2% and 3% by weight of the bitumen for better performance of mixture [15]–[18]. The Rediset content in the base bitumen was chosen as 2.0% taking the recommendation of AkzoNobel [18].

At present one type of foaming (water-containing) additive WMA technologies is Advera. Advera manufactures and markets in North America by PQ Corporation. It is powdered synthetic zeolite that has been hydro-thermally crystallized. It contains about 18–21% water of crystallization which is released by increasing temperature above 85°C. The expansion of water causes foaming of asphalt bitumen. Austerman et al. [31] and PQ Corporation [19] reported that the maximum rate of Advera in base bitumen varies between 4% and 6% by weight of bitumen. The Advera concentration in the base bitumen was chosen as 5% based on a past research made by PQ Corporation [19].

The RAP material to be utilized within warm mix asphalt was obtained from seven years old asphalt pavement. The pavement site was on the entrance of Dokuz Eylul University Tinaztepe Campus where is located on one of the main arterials in Izmir.

### Conventional Bitumen Tests

The base samples and the bitumen samples containing organic, chemical and water containing additives were subjected to the following conventional bitumen tests; penetration (ASTM D5–06), ring and ball softening point (ASTM D36–95), thin film oven test (TFOT) (ASTM D 1754–97), penetration and softening point after TFOT and storage stability test (EN 13399) [20], [21], [22]. In addition, the temperature susceptibility of the bitumen samples has been calculated in terms of penetration index (PI) using the results obtained from penetration and softening point tests [33].

The viscosity is one of the most important rheological properties of fluid that is defined as resistance to flow [34]. The effect of viscosity on bitumen’s workability is very important in selecting proper mixing and compacting temperatures. Brookfield viscometer was employed to inspect the mixing and compaction temperatures in according to ASTM D4402–06 [35]. Approximately 30 gr. of bitumen was heated in an oven so that it was sufficiently fluid to pour into the sample chamber. The amounts of bitumen varied with the different sizes of spindles. The sample chamber containing the bitumen sample was then placed in the thermo container. After the desired temperature was stabilized for about 30 min, the spindle was lowered into the chamber to evaluate the viscosity [36]. The test was performed at 135°C and 165°C. The temperatures corresponding to bitumen viscosities 170±20 mPa.s and 280±30 mPa.s were chosen as mixing and compaction temperatures respectively.

### Determining Properties of RAP

In order to obtain aged bitumen from RAP, the oxidized sample was firstly placed in reservoir of extractor. The extraction process began by placing a specified amount the RAP in the extraction vessel with a specified amount of toluene. A motor was attached and rotate the vessel for a specified time with the amount of added toluene. This was allowed the toluene/bitumen mixture to flow into the first holding flask.

Each of 1000 grams, ten batches of RAP were prepared and extraction test was performed on each of the batch to determine the bitumen content of RAP. For this test a centrifuge extractor called Rota Test was utilized.

In order to characterize the properties of the old bitumen obtained from the extraction test, conventional bitumen test methods such as: penetration test (ASTM D5–06), softening point test (ASTM D36–95), thin film oven test (ASTM D 1754–97) etc. were performed [20], [21], [22]. Following the characterization of the old bitumen, sieve analysis test were performed on the extracted aggregates.

### Mechanical Properties

The effect of RAP on the mechanical properties of WMA has been determined by the Marshall method (ASTM D3549) in terms of stability, flow and air void content as well as by the Indirect Tensile Strength (ITS) test (ASTM D6931–12) [37], [38]. The ITS test was performed by loading the specimens at a constant rate (50 mm/min vertical deformation at 25°C) and the force required to break the specimen was measured.

The tests were conducted on WMA samples containing different percentages of RAP and on samples prepared without RAP contents. Asphalt concrete specimens were prepared with a compaction effort of 75 blows simulating the heavy traffic loading conditions.

### Cost Benefit Analysis

Different techniques of producing WMA promise various energy savings for production. This mostly depends on how much the production temperature is lowered and what kind of WMA additive is used compared to HMA. The economical benefit from energy savings should be discussed together with the cost as higher energy prices promise greater savings. The cost analysis calculations are carried out in three steps. These are calculation of benefits, calculation of cost and determination of final cost.

Cost-benefit analysis was performed to inspect the advantages and disadvantages of RAP in terms of economy. For this purpose a highway section (1 km. in length, 10 m. in width and 5 cm. in thickness) is chosen. Transportation distance constitutes main part of the analysis. Therefore, the place of refinery and plant must be determined for exact analysis. For all cases, the refinery is chosen as Aliağa Refinery and the plant site is chosen as Ege Asfalt where is located in Pınarbaşı/İZMİR. The distance between the two locations is approximately 65 km. Chosen location is the center of İzmir region (Konak), the distance from Konak where RAP is taken to Ege Asfalt plant is approximately 20 km. The distance from Ege Asfalt plant to construction site is designated as M. The unit costs related to the benefits and costs of asphalt are taken from The Unit Price List of the Directorate of the General Directorate of State Highways for the year 2012. Besides, the unit costs related to three types of WMA additives are obtained from the suppliers [39]–[41].

## Results and Discussions

### Conventional Bitumen Test Results

The conventional properties of the bitumen prepared with organic, chemical and water containing additives are presented in Table 3 as a decrease in penetration and increase in softening point. Six replicates of each WMA samples were prepared for bitumen testing. The coefficient of variation (which is calculated as the ratio of standard deviation to mean value) related to bitumen tests such as penetration, softening point, viscosity, etc., varies between 0.46% and 1.58% indicating a reasonable consistency.

**Table 3:** Conventional properties of bitumen prepared with warm mix asphalt additives.

Property	Base Bitumen	Organic Additive Content	Chemical Additive Content	Water Containing Content
		(%)	(%)	(%)
		3%	2%	5%
Penetration (1/10 mm)	55	37	44	52
Softening Point (°C)	49.1	69.3	56.7	56
Penetration Index (PI)	−1.20	1.95	0.04	0.27
Retained Penetration after TFOT (%)	25	13	16	16
Softening Point Difference after TFOT (°C)	5	4	2.5	4.1
Storage Stability (°C)	–	1.6	0.5	1.6
Viscosity at 135°C (Pa.s)	0.413	0.288	0.338	0.313
Viscosity at 165°C (Pa.s)	0.138	0.075	0.087	0.112

Pavements have always been affected by susceptibility for permanent deformation or rutting caused by applied wheel loads [42]. The increase in softening point is favorable since bitumen with higher softening point may be less susceptible to permanent deformation (rutting) [43]. Organic, chemical and water containing WMA additives reduce temperature susceptibility (as determined by the penetration index-PI) of the bitumen. Lower values of PI indicate higher temperature susceptibility. Asphalt mixtures containing bitumen with higher PI are more resistant to low temperature cracking as well as permanent deformation [43].

The additives also reduce the viscosity of bitumen. This indicates that, all warm mix asphalt additives increase the workability and make relatively reductions for mixing and compaction temperatures.

**Figure 1:**
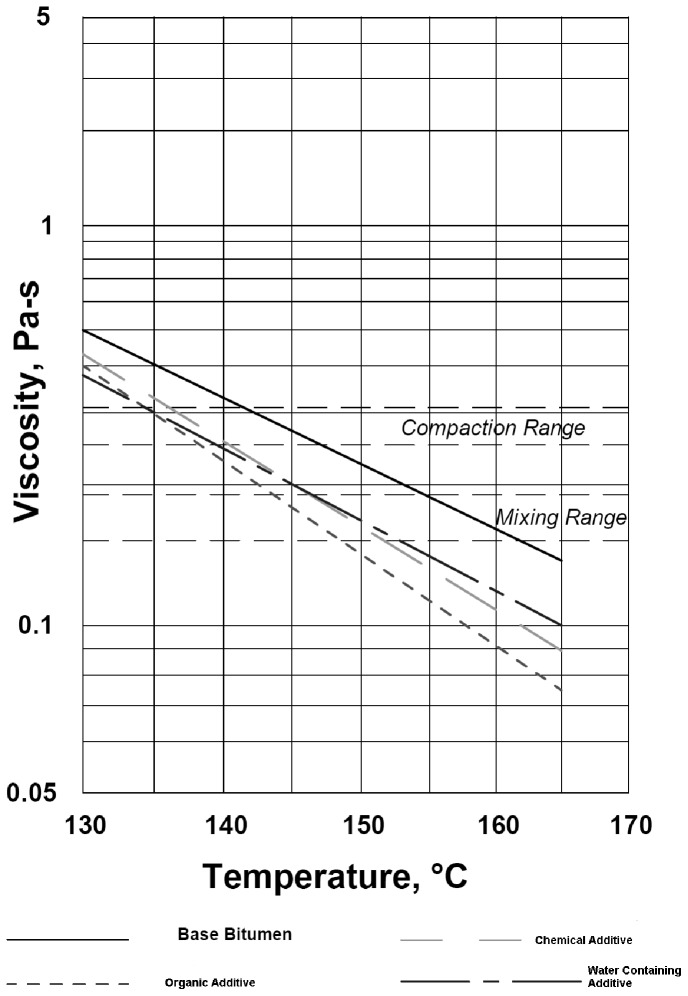
Brookfield viscometer tests results for each additive.

The results of viscosity related to each WMA additive at 135°C and 165°C are drawn at semi logarithmic figure presented in Fig. 1. The temperature that corresponds to compaction and mixing range is also summarized in Table 4.

**Table 4:** Mixing and compaction temperatures.

ADDITIVES	DOSAGE OF ADDITIVES (%)	TEMPERATURES (°C)	
		*Mixing*	*Compaction*
Base Bitumen	0	155–163	142–148
Organic Additive	3	144–149	134–138
Chemical Additive	2	147–152	136–140
Water Containing Additive	5	147–152	135–140

It is evident that the addition of organic additive reduces the mixing and compaction temperature by 13°C and 9°C respectively in comparison with the base bitumen. The addition of chemical additive reduces the mixing and compaction temperature by 10°C and 7°C. Similarly, the addition of water containing additive reduces both the mixing and compaction temperature by 9°C.

### Determining Properties of RAP

Based on the extraction test results, the average bitumen content was found as 4.30% related to ten batches of RAP samples. Conventional bitumen tests result conducted on the old bitumen is presented in Table 5.

**Table 5:** Properties of the old bitumen.

Test	Specification	Results
Penetration (25°C; 0.1 mm)	ASTM D5	23
Softening Point (°C)	ASTM D36	72.9
Penetration Index (PI)		1.45
Viscosity at (135°C)-Pa.s	ASTM D4402	0.563
Viscosity at (165°C)-Pa.s	ASTM D4402	0.138
Thin Film Oven Test (TFOT) (163°C; 5 hr)	ASTM D1754	–
Change of Mass (%)		0.02
Retained Penetration (%)	ASTM D5	18
Softening Point Diff.after TFOT (°C)	ASTM D36	1.9

As RAP bitumen reacts and loses some of its components during the construction process (short term aging) and service life of the road (long term aging), its rheological behaviour will naturally differ from virgin materials. During aging process, bitumen is exposed to hot air at high temperatures ranging from 135°C to 165°C, results in a significant increase in viscosity. Besides, bitumen loses many of its oil components during construction and service resulting in a high proportion of asphaltenes in the blend, which leads to increased stiffness and viscosity.

Sieve analysis was performed on the extracted aggregates is presented in Table 6. The mix gradation (10%, 20%, 30%, 40% and 50% of the RAP and 90%, 80%, 70%, 60% and 50% of new aggregate) must meet the requirements of Turkish Specifications related to the Type I Wearing Course construction.

**Table 6:** Sieve analysis results for extracted aggregates.

Sieve No	Cumulative Weight Passing (gr)	% Retained	% Pass
3/4″	13299	0	100
1/2″	13092	1.6	98.4
3/8″	11966	10.1	89.9
No.4	7197.5	45.9	54.1
No.10	4016	69.8	30.2
No.40	1792.5	86.5	13.5
No.80	1173	91.18	8.82
No.200	775	94.17	5.83

### Mechanical Properties

In this study, the optimum bitumen content related to WMA including organic additive, chemical additive and water containing additive were determined (by the Marshall analysis) as 4.30%, 4.53% and 4.50% respectively.

The bitumen content needed for the mix gradation of RAP and the new aggregates can be calculated by the equation: Pr  =  Pc−(Pa*Pp) where Pr is percent of bitumen to be added in the mix including RAP, Pa is percent of aged bitumen in the mix determined by Marshall test, Pc is percent of total bitumen in the mix and Pp is percent of RAP in the mix.

After determining the contents of the new bitumen to be added into mixture with respect to the values given in Table 7, the asphalt concrete samples including three different kinds of WMA additives and different percentages of RAP were prepared taken into the mixing and compaction temperatures into consideration.

**Table 7:** Calculation of the percentage of the bitumen to be added in the mix based on RAP content for each of the additive.

ADDITIVES	RAP Content	Pc (%)	Pa (%)	Pr (%)
	(%)	Total bitumen in the mix	Bitumen content of RAP	Bitumen content to be added into the mix
***Organic***	10	4.3	4.3	3.87
	20	4.3	4.3	3.44
	30	4.3	4.3	3.01
	40	4.3	4.3	2.58
	50	4.3	4.3	2.15
***Chemical***	10	4.53	4.3	4.10
	20	4.53	4.3	3.67
	30	4.53	4.3	3.24
	40	4.53	4.3	2.81
	50	4.53	4.3	2.38
***Water Containing***	10	4.50	4.3	4.07
	20	4.50	4.3	3.64
	30	4.50	4.3	3.21
	40	4.50	4.3	2.78
	50	4.50	4.3	2.35

**Figure 2:**
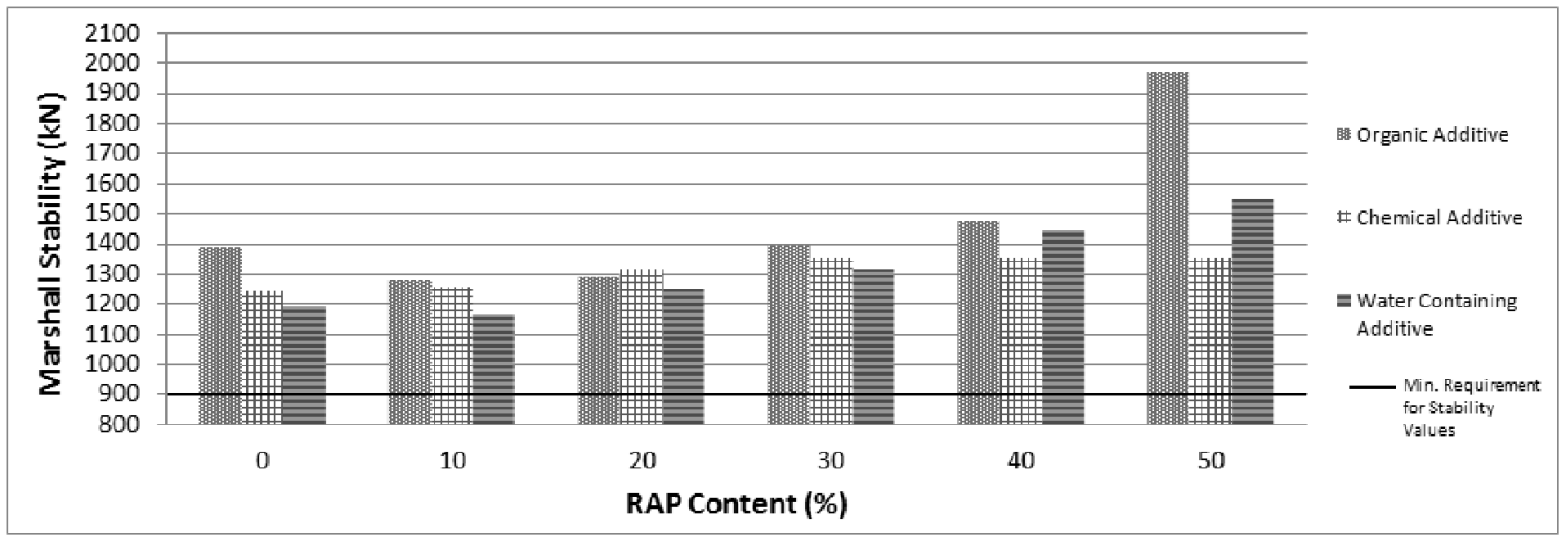
Marshall stability values for RAP and control samples.

**Figure 3:**
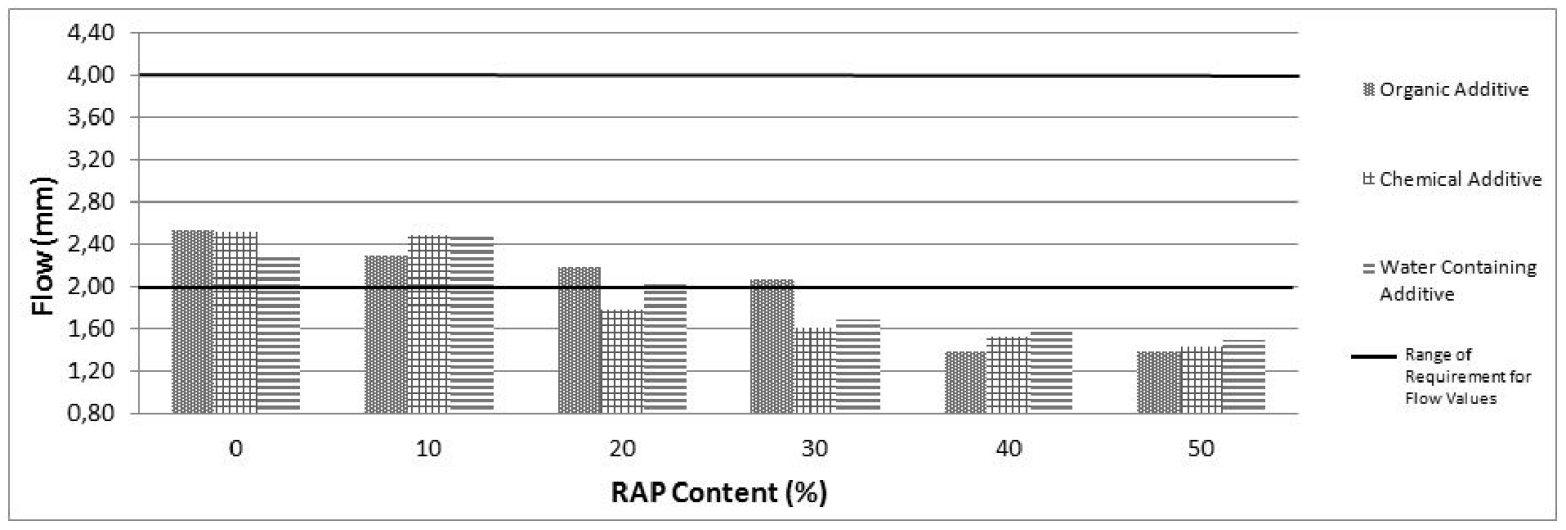
Flow values for RAP and control samples.

**Figure 4:**
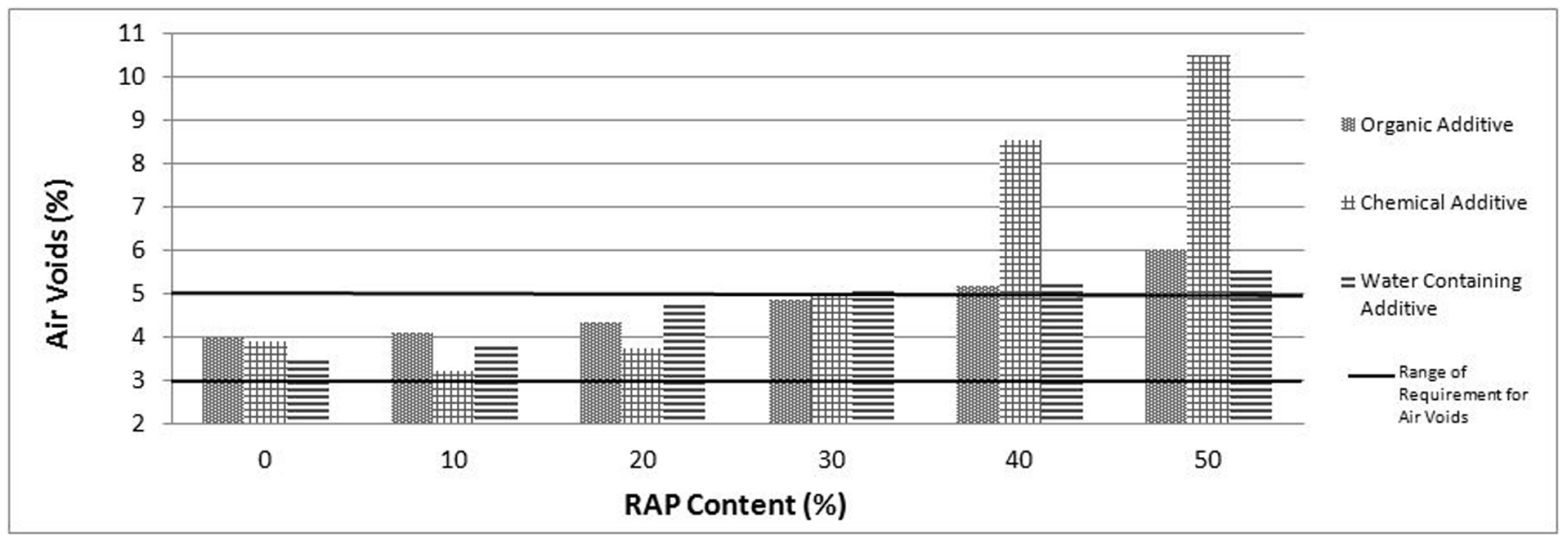
Air void values for RAP and control samples.

The mechanical properties of different RAP percentages with all warm mix additives in terms of stability, flow and voids content are presented in Fig. 2, Fig. 3 and Fig. 4 respectively.

As illustrated in Fig. 2, all recycled asphalt mixtures involving all WMA additives provide adequate stability (min. 900 kg. related to wearing course specification). The stability values increase with increasing of RAP contents for the mixtures prepared with organic additive and water containing additive. However, no significant variation is observed on the stability values above 30% RAP content addition for the mixtures involving chemical additive. As presented in Fig. 3; the flow values decrease with increasing RAP contents for the mixtures prepared with all WMA additives. As the flow values are indicator of deformation characteristic, the flow values are less than the specification limits (2 mm.) is not favourable since it implies that the mix is very stiff and brittle. As depicted in Fig. 3, more than 30%, 10% and 20% RAP addition are below the specification limits of flow values for mixtures prepared with organic, chemical and water containing additive respectively. Therefore, it can be concluded that the 30% RAP content with organic additive, 10% RAP content with chemical additive and 20% RAP content with water containing additive can be accepted as an optimum RAP content based on the specification limits of flow and stability values.

As illustrated in Fig. 4, as RAP contents increase, the voids increase as well for all specimens involving WMA additives due to crystallize structure of oxidized RAP materials. Besides, the concluded optimum RAP contents for each WMA additive satisfy the specification limits of air voids value (3%–5%).

**Figure 5:**
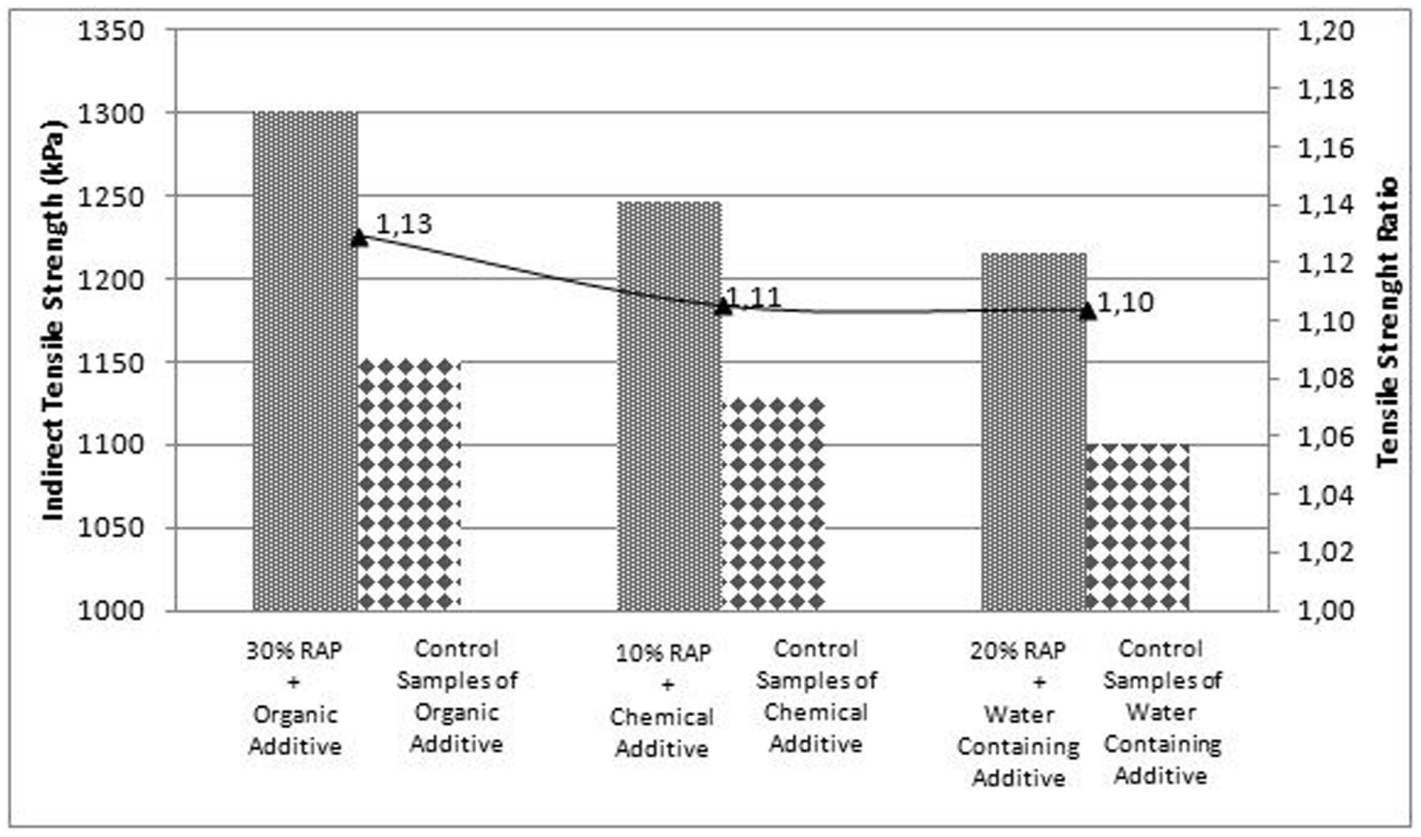
ITS results of control samples and optimum RAP contents for each WMA additives.

Indirect Tensile Strength (ITS) test results of control samples (without RAP) and samples involving optimum RAP contents with WMA additives are presented in Fig. 5. The tensile strength ratio (the ratio of ITS of sample with RAP to ITS of control sample) is also presented in the same figure.

As seen in Fig. 5, the ITS results of mixtures involving RAP are higher than the control samples. The increase in ITS values of mixtures can be attributed to the increased stiffness of the mixtures involving RAP. The greater tensile strength of the mixture containing RAP as compared to control mixture also indicates greater cohesive strength of the WMA with RAP.

Among the utilized WMA additives together with optimum RAP addition, WMA mixture involving organic additive with 30% RAP depicted the most tensile strength exhibiting the tensile strength ratio as 1.13.

The variance values of asphalt concrete stability test which is related to WMA mixtures prepared with different additives and different percentages of RAP lies between 171 and 231, which corresponds to deviation ratio of 0.98%–1.14%. The variances related to flow values of WMA mixtures prepared with different percentages of RAP lies between 3.2E-04 and 3.46E-04 which corresponds to deviation ratio of 0.78–0.82%. The above calculated variances indicate that the determined results of stability and flow vary in acceptable ranges.

### Evaluation of Cost Benefit Analysis

Following the determination of the optimum RAP content for each WMA additive, cost-benefit analysis was performed to inspect the advantages of RAP in terms of economy.

**Figure 6:**
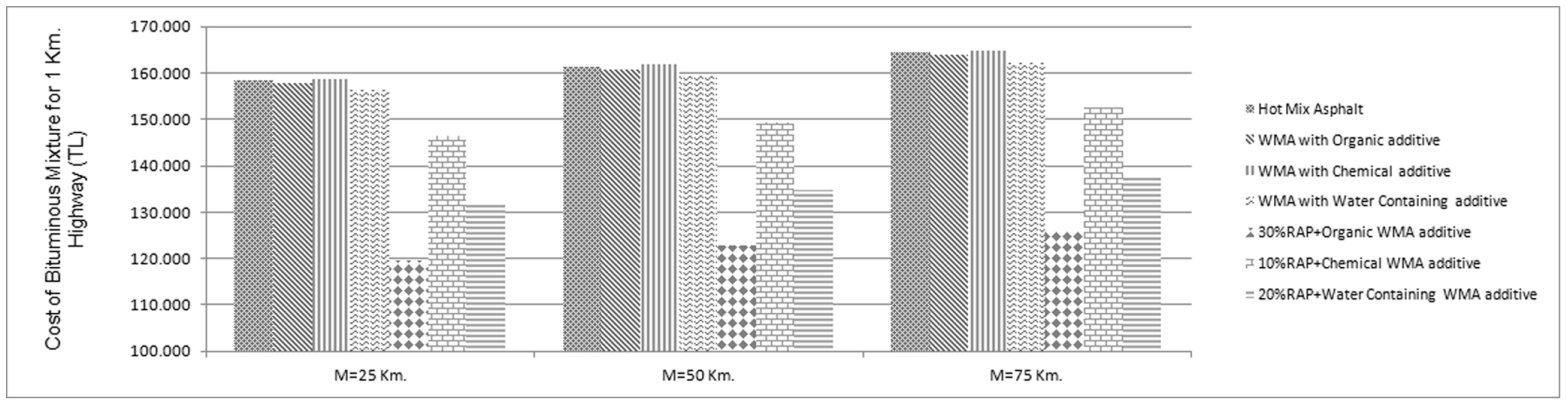
Illustration of cost analysis results.

The calculation of cost analysis conducted on HMA, WMA and an optimum RAP content in terms of M (distance from plant to construction site) is presented in Fig. 6 and each details during the calculation is given in Table 8.

**Table 8:** Cost-benefit analysis results.

	HOT MIXASPHALT	WARM MIXASPHALT			WARM MIXASPHALT+RAP		
		OrganicWMAAdditive	ChemicalWMAAdditive	WaterContainingWMA Additive	30% RAP+OrganicAdditive	10% RAP+ChemicalAdditive	20% RAP+WaterContaining Additive
***Total aggregate cost*** ***(TL/ton)***	65.77	65.77	65.77	65.77	46.039	59.193	52.616
***Total bitumen cost*** ***(TL/ton)***	60.3778	53.202	56.047	55.676	37.24	50.727	45.0359
***The cost of bitumen transportation*** ***from the place of*** ***delivery to storage tank (TL/ton)***	0.392718	0.346042	0.3646	0.3621	0.242	0.3299	0.293
***The cost of bituminous mixture*** ***transportation from plant to*** ***construction site(TL/ton)***	0.1015*M +1.45	0.1015*M +1.45	0.1015*M +1.45	0.1015*M +1.45	0.1015*M +1.45	0.1015*M +1.45	0.1015*M +1.45
***The cost of bituminous adhesive*** ***agent transportation from*** ***Aliağa Refinery to Ege Asfalt(TL/ton)***	0.0338	0.0338	0.0338	0.0338	0.0338	0.0338	0.0338
***Heating of the bitumen*** ***(TL/ton)***	1.304912	1.0352	1.109	1.12833	0.7246	1.00412	0.9127
***Cost of WMA additive*** ***(TL/ton)***	–	7.02	4.93	3.19	4.914	4.462	2.584
***Cost of RAP excavation*** ***(TL/ton)***	–	–	–	–	5.469	1.823	3.646
***Cost of RAP transportation*** ***(TL/ton)***	–	–	–	–	1.044	0.348	0.696
***Cost of 1 tone bituminous*** ***mixture (TL)***	129.329+0.1015*M	128.857+0.1015*M	129.704+0.1015*M	127.610+0.1015*M	97.156+0.1015*M	119.371+0.1015*M	107.267+0.1015*M
***Cost of bituminous mixture for*** ***1 km. highway (TL)***	155.195+121.8*M	154.629+121.8*M	155.645+121.8*M	153.132+121.8*M	116.585+121.8*M	143.245+121.8*M	128.721+121.8*M
***Case study of bituminous*** ***mixture for 1 km. highway*** ***(TL), M = 25 km.***	**158.240**	**157.674**	**158.690**	**156.177**	**119.633**	**146.290**	**131.766**
***Case study of bituminous*** ***mixture for 1 km. highway*** ***(TL), M = 50 km.***	**161.285**	**160.719**	**161.735**	**159.222**	**122.678**	**149.335**	**134.811**
***Case study of bituminous*** ***mixture for 1 km.*** ***highway (TL), M = 75 km.***	**164.330**	**163.764**	**164.780**	**162.267**	**125.723**	**152.380**	**137.856**

An initial comparison was made between hot mix and warm mix asphalt. For all M values, organic additive reduces the final cost. However, the similar conclusion cannot be made for chemical additive.

On the occasion when RAP is taken into consideration, as expected the utilization of RAP decreases the final cost for all cases. Among the RAP additions, it is clearly observed that utilizing of 30% RAP content with organic additive is the most economic in terms of final cost for all case studies that are calculated for various distances (M  = 25 km., 50 km. and 75 km.) from plant to construction site.

The road industry has been seeking to minimize the amount of energy required to produce asphalt mixture and to lower asphalt plant emissions, parallel to energy savings and environmental benefits for many years.

Recycling processes save energy. Saved of aggregates reduces necessities of quarrying, transportation and the subsequent processing in recycling methods. Consequently, cost of energy is saved in these processes. Recycled asphalt reduces the demand for new bitumen and saves energy at the refinery. Moreover, electric power consumption significantly decreases because of reduced demand for bitumen.

Emissions from HMA are harmful to the environment during the laying and compaction steps. The emissions in HMA include nitrogen oxides, carbon monoxide, sulphur dioxide and the other volatile organic components. The organic WMA additive, and construction temperatures affect on carbon dioxide emissions. This result means that carbon dioxide emission depends on temperature. Thus, decreasing of asphalt mixing or compaction temperatures is a way to decrease amount of carbon dioxide emissions during pavement construction.

Additional important benefit of the WMA technology is the reduction of energy consumption required by heating in traditional HMA to typically found at the production plant. With the decreased production temperature, occurs the additional benefit of reduced emissions at the plant and during lay down. Fuel savings with WMA typically range from 20 to 30%. These rates can be higher than 50% or more in the processes with low energy concrete. The reduced fuel and energy usage give a reduction of the production of green house gases and reduces the carbon footprint.

## Conclusions and Recommendations

Lowering asphalt production emissions and compaction emissions in the plant are the most important benefits of utilization of warm mix asphalt. The properties of bitumen are improved by means of organic, chemical and water containing WMA additives. These results have been reached by the conventional bitumen test methods such as penetration, softening point, rotational viscosity, TFOT test results. Besides, the utilization of organic, chemical and water containing additives help in the reduction of viscosity values which are in return decreases the mixing and compaction temperature leading to the reduction of energy costs as well as emissions.

Marshall Stability values related to RAP mixtures have been found higher than the control mixtures. Based on the utilized aggregate, 30%, 10% and 20% can be accepted as an optimum RAP addition related to organic, chemical and water containing additives respectively. The other properties of samples including optimum RAP content for each used additive such as flow, air void level are also within the specification limits. The utilization of RAP with WMA exhibits low flow values with high stability values and hence high Marshall Quotient (MQ) values are indicating a high stiffness mixture with a greater ability to spread the applied load and resist creep deformation. Care must be exercised with very high stiffness mixes due to their lower tensile strain capacity to failure; such mixes are more likely to fail by cracking particularly when laid over foundations which fail to provide adequate support.

Indirect tensile strength (ITS) is a very common performance test used in pavement industry. ITS testing offers a reliable indication of the crack potential for a mixture. Organic additive with 30% RAP content has an appreciable increase in tensile strength over the control mix, which may be due to crystallize structure of both organic additive aided WMA mixture and RAP materials.

The main benefit of the RAP with WMA technology is the ability to reduce final cost compared to HMA and WMA mixtures. The reduction rate is strongly connected with the less need of virgin bitumen, virgin aggregates and less need of heating process that are used in WMA mixtures containing with RAP. Among the RAP additions, it is clearly observed that utilizing of 30% RAP content with organic additive is the most economic in terms of final cost for all case studies.

The conclusion of the study covers the utilization of three types of warm mix asphalt additives with different percentages of RAP materials with 50/70 penetration grade base bitumen.

Moisture susceptibility is an important issue for WMA mixtures including RAP that enable low mixing, laying and compaction temperatures compared to conventional HMA. If the aggregate is not dried prior to mixing, the inherent moisture can prevent the bitumen from bonding with the surface of the aggregate, which may lead to stripping. Besides, it is known that the oxidized RAP material adversely affects the cohesion and more significantly adhesion mechanism of the bitumen- aggregate interface system. More research can be conducted to evaluate the stripping resistance of WMA mixtures including RAP by way of performing Modified Lottman test. The long term performance evaluations and more extensive tests may be performed containing different WMA additives and different penetration grade bitumen.
